# Messages matter: The Tobacco Products Directive nicotine addiction health warning versus an alternative relative risk message on smokers' willingness to use and purchase an electronic cigarette

**DOI:** 10.1016/j.abrep.2018.09.006

**Published:** 2018-09-21

**Authors:** Sharon Cox, Daniel Frings, Reeda Ahmed, Lynne Dawkins

**Affiliations:** Centre for Addictive Behaviours Research, School of Applied Sciences, London South Bank University, 103 Borough Road, London SE1 0AA, UK

**Keywords:** E-cigarettes, Smoking, Nicotine, Health messages, Health warning

## Abstract

**Introduction:**

Many countries have now mandated warning labels on e-cigarette products. One example, the EU TPD health warning states, “This product contains nicotine which is a highly addictive substance. [It is not recommended for use by non-smokers]”. The impact of the EU TPD warning message on intentions to use, has not been explored within an EU population.

**Aims:**

Examine the effect of i) the TPD e-cigarette health warning and ii) an alternative relative risk message, on smokers' willingness to use, likelihood of purchase, and intention to use as a quit aid.

**Methods:**

Cross-sectional online study. Ninety-five smokers (55 males; 18–55 years old) were randomly allocated to one of three conditions and viewed ten individually presented e-cigarettes images with either no message, TPD message, or relative risk message. Participants rated i) willingness to use, and likelihood of: ii) purchase, iii) using in the next month, and iv) using in a quit attempt, before and after viewing the images.

**Results:**

For willingness to use and likelihood of purchase, ANCOVAs showed a significant main effect of Message Type (*p*s, <.05); ratings were lower in the TPD condition. Message type, however did not significantly change likelihood of using in the next month or using in a quit attempt.

**Conclusions:**

Preliminary findings suggest that the TPD e-cigarette health warning may reduce smokers' willingness to use and likelihood of purchasing an e-cigarette. Messages conveying reduced harm or indeed, no message at all, may be more effective in encouraging smokers to switch to these lower risk products.

## Highlights

• Examine the effect of i) the TPD e-cigarette health warning and ii) an alternative relative risk message
• TPD e-cigarette health warning may negatively affect smokers' willingness and intentions to use.
• Messages conveying reduced harm may be more effective in encouraging smokers to switch to reduced harm products.

## 1 Introduction

Electronic cigarettes (e-cigarettes) are a tobacco harm reduction product generally agreed to be far less harmful than smoking with some leading health organisations estimating that they carry around 5% the health risk of tobacco smoking (McNeill, Brose, Calder, Bauld, & Robson's, 2018, Public Health England (PHE); RCP, 2016). In the UK, an estimated 2.9 m smokers have quit smoking using e-cigarettes (Action on Smoking and Health (ASH, 2017)) and smoking cessation is the most commonly cited reason for use (Office for National Statistics, 2016). E-cigarettes, therefore, have potential to aid smoking cessation and reduce smoking related disease and evidence suggests they are as, or more, effective than NRT (Brown, Beard, Kotz, Michie, & West, 2014; McRobbie, Bullen, Hartmann-Boyce, & Hajek, 2012), though there is evidence from England and other EU countries that many smokers who use e-cigarettes also continue to smoke (West, Beard, & Brown's, 2018, Smoking Toolkit Study (STS); Farsalinos et al., 2018).

Despite the growing prevalence of e-cigarettes, especially in the UK (up until 2016; West et al., 2018), in recent years the public's perception pertaining to harms related to e-cigarettes have increased. For example, in one survey in Great Britain (ASH, 2017) only 13% of respondents correctly believed that e-cigarettes are considerably less harmful than tobacco smoking (ASH, 2017). Similar results have also been found in a US sample (Majeed et al., 2017). Reasons for these misperceptions may include a general misunderstanding of the harms of nicotine use, as well as the wider impact of negative media reporting (McNeill et al., 2018). It is possible that health warnings on e-cigarettes may exacerbate these misperceptions by negatively impacting smokers' beliefs and acting as a deterrent to use in a quit attempt (Wackowski, Hammond, O'Connor, Strasser, & Delnevo, 2016).

Many countries have now mandated warning labels on e-cigarette products. These are typically borrowed or amended messages from cigarette or smokeless tobacco products and often refer to nicotine. Article 20 the EU Tobacco Products Directive [TPD] (20th May 2016) stipulates that e-cigarette packets and refill products must carry a health warning covering 30% of the packaging, either: i) ‘*This product contains nicotine which is a highly addictive substance. It is not recommended for use by non-smokers*’ or ii) ‘*This product contains nicotine which is a highly addictive substance*’. Such warning labels may be especially effective in deterring non-smokers from trying an e-cigarette. Supporting this, two recent studies demonstrated that perceived harm, addictiveness and intention to use in US and Canadian young adult non-smokers declined following exposure to nicotine addiction health warnings (Czoli, Goniewicz, Islam, Kotnowski, & Hammond, 2015; Mays, Smith, Johnson, Tercyak, & Niaura, 2016).

Whilst reducing e-cigarette appeal among non-smokers is clearly desirable, as noted above, this may have the unintended consequence of reducing appeal among smokers who may be considering e-cigarette use for smoking cessation (Berry, Burton, & Howlett, 2017). Research on e-cigarette health warnings is limited to date and has been concentrated in the US and Canada. In a nationally representative sample of US adults, exposure to e-cigarette magazine adverts containing a negatively framed health warning did not increase the probability of rating e-cigarettes to be more or equally harmful compared to cigarettes, particularly in non-smokers (Shang et al., 2018). However, findings from six small focus groups with e-cigarette users and smokers suggests that health warnings including statements that e-cigarettes can be poisonous, contain toxins or are “not a safe alternative to smoking” could reduce appeal among smokers who may be considering e-cigarettes for smoking cessation (Wackowski et al., 2016). E-cigarette advertisements that include an addiction warning also increased health-risk beliefs in smokers and e-cigarette users, which in turn, negatively influenced willingness to try the product (Sanders-Jackson, Schleicher, Fortmann, & Henriksen, 2015). Conversely, positively framed advertising messages that focused on differences between cigarettes and e-cigarettes (e.g. healthier, helps to quit smoking) rather than similarities (feels like smoking, relieves cravings) created more interest among smokers in trying an e-cigarette (Pepper, Emery, Ribisl, Southwell, & Brewer, 2014). Messages conveying reduced harm information have also been associated with lower odds of immediate smoking and may therefore encourage smoking cessation (Jo, Golden, Noar, Rini, & Ribisl, 2018).

To date, whilst the impact of health warning labels have been explored, the specific impact of EU TPD e-cigarette health warning label on intentions to use has not been explored. We will test the hypothesis that the TPD e-cigarette nicotine addiction health warning reduces i) willingness to use, ii) likelihood of purchasing, iii) likelihood of using in the next month and iv) likelihood of using in a quit attempt among smokers. Importantly, we also explore whether viewing an alternative relative risk message (i.e. evidencing that e-cigarettes are less harmful than tobacco smoking) can increase smokers' willingness to use, likelihood of purchasing, using in the next month, and using in a quit attempt.

## 2 Methods

### 2.1 Participants and procedure

One-hundred daily smokers responded to adverts on a student focused Research Participation Scheme (RPS) or Facebook by clicking on a link directing them to Qualtrics. Five incomplete responses and 14 participants who described themselves as daily e-cigarette users were removed leaving 81 participants (45 males) aged between 18 and 55 (*M =* 25.06, *SD* = 7.12) Following informed consent they completed baseline questions relating to demographics, smoking and e-cigarette history, motivation to quit smoking, nicotine dependence and intentions and willingness to use e-cigarettes. Participants were then then randomly allocated to one of three conditions: TPD health message, a reduced harm message or no message. All three conditions viewed ten separate images of electronic cigarettes (identical across conditions) in the same sequence with either the presence or absence of the health message (according to condition). In both message conditions, for each e-cigarette image, the message was presented in black Arial font against a white background and occupied 15% of the screen. Participants could view the images for as long they wished and simply pressed click, to move to the next image. Thereafter participants once again completed ratings of intention and willingness to use e-cigarettes before debriefing. Students were offered 2 RPS course credits for their participation. Ethical approval was gained from the university ethics committee.

### 2.2 Materials

Participants provided information on current smoking habits (smoking length, cigarettes per day, last quit attempt, length of quit attempt), motivation to stop smoking (MTSS; Kotz, Brown, & West, 2013) and nicotine dependence (Fagerström Test of Cigarette Dependence [FTCD]; Heatherton, Kozlowski, Frecker & Fagerstrom, 1991).

Ten e-cigarettes images (including a combination of cigalikes, second and third generation devices) were presented individually with either no message, the TPD message (“This product contains nicotine which is a highly addictive substance. It is not recommended for use by non-smokers”) or a reduced-risk message (“The Royal College of Physicians (2016) report concluded that e-cigarettes are 95% less harmful than cigarettes”) according to allocated condition.

Intention and willingness to use was measured using the following questions. 1) How willing would you be to use an e-cigarette? 2) How likely is it that you will purchase an e-cigarette in the next month? 3) How likely is it that you will use an e-cigarette in the next month? 4) How likely is it that you will use an e-cigarette in a serious attempt to quit smoking? Each response was measured on a 7 point Likert scale (from 1 = not at all likely to 7 = extremely likely). Current e-cigarette use was determined using the question: How often, do you use an electronic cigarette? (a) never; (b) once or twice, (c) weekly and (d) daily.

## 3 Results

### 3.1 Smoking, e-cigarettes use and cessation

Mean FTCD scores were 2.42 (SD = 2.47) and participants reported smoking for an average of 8.83 years (*SD* = 7.13) and on average, 9.63 (*SD* = 5.91) cigarettes per day (CPD). 33.3% of participants reported attempting to quit in the last year, 24.7% within the last 6 months, 13.6% within the last month and 7.4% in the last week; 21% reported never attempting to quit. 32.1% reported their quit attempt lasting one month, 22.2% lasting one week, 22.2% lasting one day, 9.9% lasting 6 months and 7.4% lasting one year. 34.6% described themselves as knowing they should stop smoking but not wanting to. Among the participants 72.8% had never used an e-cigarette and 27.2% had used once or twice.

### 3.2 Randomisation checks

To check randomisation had been successful, a multiple ANOVA was conducted with Message Type (TPD message, reduced harm message, no message) as the independent variable and age, gender, FTCD, motivation to quit, length of smoking, e-cigarette use, cigarettes smoked per day, time since last quit attempt and duration of last quit attempt. Randomisation to condition failed for duration of last quit attempt (*p* < .05). FTCD scores and length of smoking also differed marginally as a function of condition (*p* = .069 and 0.77 respectively). As such, these factors were included in our main analysis. Other factors were successfully randomised (*p*s > .341).

### 3.3 Main analysis

To test the effects of Message Type on willingness to use, likelihood of purchasing, likelihood of using in the next month and likelihood of using in a quit attempt, a series of ANCOVAs were performed. These included Time 1 ratings, and the covariates listed above. For willingness to use and likelihood of purchase, these ANCOVAs showed a significant main effect of Message Type (*p*s, <.05). For likelihood of using in the next month and likelihood of using in a quit attempt, there was no significant change. Full details of these ANCOVAs can be found in Table 1. Post hoc comparisons on covariate adjusted means were undertaken (unadjusted means are reported in Table 2).

**Table 1:** Results of ANCOVAs testing the effects of Message Type on intentions.

Intention	Message type			Covariates											
				Time 1 rating			FTCD total			Length last quit			Years of smoking		
	*F*	*p*	η_p_^2^	*F*	*p*	η_p_^2^	*F*	*p*	η_p_^2^	*F*	*p*	η_p_^2^	*F*	*p*	η_p_^2^
Willing to use	4.72	.012	0.13	65.63	<.001	0.51	0.001	.978	<0.01	0.098	.755	0.002	5.38	.02	0.08
Purchase	4.54	.014	0.12	185.34	<.001	0.74	2.02	.160	0.03	0.095	.759	<0.01	0.534	.468	<0.01
Use in next month	1.82	.17	0.05	77.54	<.001	0.55	2.06	.156	0.03	0.016	.901	<0.01	0.204	.653	<0.01
Use as a quit-aid	2.20	.12	0.06	147.79	<.001	0.70	0.001	.804	<0.01	0.31	.583	<0.01	0.62	.804	<0.01

Note: For all ANCOVAs covariate dfs were (1,64) and Message Type dfs were (2,64).

**Table 2:** Mean post message exposure e-cigarette intentions as a function of Message Type. Standard deviation in parentheses. Higher scores indicate more likely.

Intention	Message Type condition		
	TPD	No message	Reduced harm
Willingness to use	2.07 (1.46)^ab^	3.22 (1.91)^a^	2.95 (1.79)^b^
Purchase	1.82 (1.61)^a^	2.00 (1.31)^a^	2.15 (1.31)
Use in next month	1.82 (1.47)#	2.48 (1.78)	2.45 (1.78)#
Use as quit aid	2.43 (1.87^)⁎^	3.13 (1.96)⁎	2.80 (1.85)

Note: Means sharing a superscript within a row differ significantly, *p <* *.*05*.*

⁎ *p* = .065.

# *p* *=* .080.

## 4 Discussion

In this sample of 81 smokers who viewed e-cigarette images accompanied by either the TPD message, a reduced harm message or no message, ratings of willingness to use and likelihood of purchase were significantly lower after viewing the TPD nicotine addiction message. The same pattern of results was found for likelihood of using in the next month and likelihood of using in a quit attempt although these fell short of statistical significance. Where significant effects were found between message conditions, these were generally between TPD vs. no message, or TPD vs. reduced harm message; ratings did not differ between the no message and reduced harm message conditions. These findings concur with Wackowski et al. (2016) and Berry et al. (2017) and suggest that the current TPD message (“This product contains nicotine which is a highly addictive substance. It is not recommended for use by non-smokers”) may discourage smokers from using e-cigarettes.

Although we did not measure risk perceptions, it is possible that the TPD message increased perceptions of harms associated with e-cigarette use which in turn reduced willingness and likelihood of purchasing/using. The emphasis on nicotine addiction may further fuel concerns about maintaining addiction (ASH, 2017) or increase perceptions of the harmfulness of nicotine which has been reported elsewhere to be misunderstood and commonly confused with the harms of tobacco smoking (Byron, Jeong, Abrams, & Brewer, 2018; Moysidou et al., 2016; Smith, Curbow, & Stillman, 2007).

Our study did not include non-smokers. Deterring non-smokers is clearly an important goal of e-cigarette health messages but this needs to be carefully balanced against potential negative effects on smokers given the reduced harm status of e-cigarettes (McNeill et al., 2018; RCP, 2016) and the increasing evidence for their effectiveness as a quit aid (Brown et al., 2014; McRobbie et al., 2012). Contrary to Pepper et al. (2014) and Jo et al. (2018) we observed no effect of a reduced harm message (“The Royal College of Physicians (2016) report concluded that e-cigarettes are 95% less harmful than cigarettes”) on willingness and likelihood of purchasing/using compared with the no message condition, however, the reduced risk message did increase willingness to use compared with the TPD message. Thus there may be utility in combining a reduced risk message with the current TPD health warning in order to encourage uptake among smokers and simultaneously deter non-smokers. Exploration of the effects of such message combinations on smokers and non-smokers are certainly worthy of further empirical investigation.

There are several limitations to our study. We focused on just one reduced risk message which was not piloted for readability, source credibility and convincingness - all of which have been shown to be important factors influencing message effectiveness (Bansal-Travers, Hammond, Smith, & Cummings, 2011; Institute for Global Tobacco Control, 2013). Alternative reduced risk messages may be more appropriate. There was also no control for duration of exposure in our study and participants could decide how long to view each image. Although this scenario is more likely to reflect how potential uses may view e-cigarette images when browsing online, a degree of experimental control is lost. Our sample of smokers were predominantly (65%) students with fairly low FTCD scores suggesting a lower level of cigarette dependence; whether our findings generalise to heavier, more dependent smokers remains to be determined. Furthermore, this study was conducted in England, a country with low restrictive e-cigarette policies, findings are likely to be different in countries with stricter regulatory policies. Finally, although self-reported willingness to use and behavioural intentions may be suggestive, actual purchasing and usage behaviour was not measured.

To conclude, although our findings are preliminary and in need of replication, they suggest that the TPD nicotine addiction e-cigarette health warning may reduce smokers' willingness to use, and likelihood of purchasing an e-cigarette. Given the reduced harm potential of e-cigarettes compared with tobacco cigarettes, a better understanding of how messaging may influence product choice and quitting strategies under differing regulatory frameworks is needed.

## Author disclosures

### Role of funding

None.

### Contributors

Authors RA and LD conceptualised the project, RA was responsible for the daily running of the project including data collection. DF conducted the statistical analysis. SC and LD conducted the literature reviews and wrote the first draft manuscript. All authors contributed to and have approved the final manuscript.

### Conflicts of interest

SC has provided consultancy to the Pacific Life Insurance Group relating to reduced risk product and smoking use and prevalence rates.

DF is an investigator on a randomised controlled trial funded by Allen Carrs Easyway Ltd. This trial is comparing the Allen Carr Easyway stop-smoking method to local NHS 1–1 stop smoking counselling service. The team are contractually free to publish the results regardless of the study outcome.

RA has no competing interests.

LD has provided consultancy for the pharmaceutical industry (2015, 2017) and acted as an expert witness for an e-cigarette patent infringement case (2015). Between 2011 and 2013 she conducted research for several independent electronic cigarette companies for which the University of East London received funds. The e-cigarette companies involved had no input into the design, conduct or write up of these projects.

## Funding

This research received no specific grant from any funding agency, commercial or not-for-profit sectors.
